# Unlocking the "Black box": internal female genitalia in Sepsidae (Diptera) evolve fast and are species-specific

**DOI:** 10.1186/1471-2148-10-275

**Published:** 2010-09-10

**Authors:** Nalini Puniamoorthy, Marion Kotrba, Rudolf Meier

**Affiliations:** 1 Institute of Evolutionary Biology and Environmental Sciences, University of Zurich, Winterthurerstrasse 190, CH-8057 Zurich, Switzerland; 2 Department of Biological Sciences and University Scholars Programme, National University of Singapore, 14 Science Drive 4, Singapore 117543, Singapore; 3 Bavarian State collection of Zoology, Münchhausenstrasse 21, D-81247 Munich, Germany

## Abstract

**Background:**

The species-specificity of male genitalia has been well documented in many insect groups and sexual selection has been proposed as the evolutionary force driving the often rapid, morphological divergence. The internal female genitalia, in sharp contrast, remain poorly studied. Here, we present the first comparative study of the internal reproductive system of Sepsidae. We test the species-specificity of the female genitalia by comparing recently diverged sister taxa. We also compare the rate of change in female morphological characters with the rate of fast-evolving, molecular and behavioral characters.

**Results:**

We describe the ectodermal parts of the female reproductive tract for 41 species representing 21 of the 37 described genera and define 19 morphological characters with discontinuous variation found in eight structures that are part of the reproductive tract. Using a well-resolved molecular phylogeny based on 10 genes, we reconstruct the evolution of these characters across the family [120 steps; Consistency Index (CI): 0.41]. Two structures, in particular, evolve faster than the rest. The first is the ventral receptacle, which is a secondary sperm storage organ. It accounts for more than half of all the evolutionary changes observed (7 characters; 61 steps; CI: 0.46). It is morphologically diverse across genera, can be bi-lobed or multi-chambered (up to 80 chambers), and is strongly sclerotized in one clade. The second structure is the dorsal sclerite, which is present in all sepsids except *Orygma luctuosum *and *Ortalischema albitarse*. It is associated with the opening of the spermathecal ducts and is often distinct even among sister species (4 characters; 16 steps; CI: 0.56).

**Conclusions:**

We find the internal female genitalia are diverse in Sepsidae and diagnostic for all species. In particular, fast-evolving structures like the ventral receptacle and dorsal sclerite are likely involved in post-copulatory sexual selection. In comparison to behavioral and molecular data, the female structures are evolving 2/3 as fast as the non-constant third positions of the *COI *barcoding gene. They display less convergent evolution in characters (CI = 0.54) than the third positions or sepsid mating behavior (CI_*COI *_= 0.36; CI_BEHAV _= 0.45).

## Background

The diversity and morphology of male genitalia have been well documented in many insect taxa [1-7], and most evolutionary biologists agree that sexual selection is responsible for driving the frequent and rapid divergence of these structures [6,8-11]. In comparison to male structures, the external and internal female reproductive tract is largely a "black box" that remains poorly known. Taxonomists routinely use male but not female genitalia for species identification, and there is some evidence that female genitalia are indeed less variable than their male counterparts [12,13]. However, it is important to remember that most taxonomists are interested in studying structures that are easily accessible and preserve well under different conditions (e.g., ethanol, pinned specimens). Male genitalia, and in particular the intromittent organ, are ideal candidates, because they are external and generally well sclerotized [1,14,15]. In contrast, the part of the female reproductive tract that is interacting with the male intromittent organ and thus most likely to show species-specific differences is internal, fairly inaccessible, and membranous. It is therefore not surprising that relatively few systematists routinely study the internal female genitalia for taxonomic purposes, and much less comparative data are available for females. Here, we target the internal female reproductive tract in a comparative study across the Sepsidae (Diptera: Insecta).

The female reproductive tract has recently attracted more attention for other reasons [16-21]. It is likely involved in sexual conflict, sperm competition, and cryptic female choice [8,9,22-24] and generally acknowledged that detailed knowledge of the morphology of the reproductive organs is crucial for understanding their role in post-copulatory sexual selection [25,26]. For instance, in Diptera, some studies use morphological data for investigating post-copulatory sexual selection through female choice [e.g: Muscidae, [18], Dryomyzidae [27], and Tephritidae, [28]], sperm competition [e.g.: Drosophilidae, [11,26,29]] or sexual arms races [e.g.: Scathophagidae, [22]]. These studies indicate that females can influence paternity by differentially storing sperm from various males in separate sperm storage organs (e.g., spermathecae) and controlling which sperm is used for fertilizing eggs [19,26,30]. For example, in phlebotomine sandflies (Psychodidae), the male intromittent organ (i.e. the aedaegus) deposits sperm directly into the spermathecae, extending past the length of the spermathecal ducts [31,32]. This, however, is not the case for most Diptera where males deposit the sperm in the bursa copulatrix (i.e. the vagina) and/or close to the spermathecal duct openings [28]. Based on studies of in-copula pairs of *Microsepsis *and *Archisepsis*, this is also the case in Sepsidae, sometimes involving the formation of an internal spermatophore [33-35].

With the exception of a few [16,22,26,36], most studies of the female reproductive tract in Diptera are based on single species and comparative data across multiple species are rare. Here, we present information on a large number of species of Sepsidae, a family of flies that is frequently used in sexual selection studies. Currently, the internal female reproductive system of Sepsidae is poorly known. Early schematic depictions are superficial or even misleading in their generalizations of the female structures [37-39]. More recently, there is photographic documentation in Kotrba [40] for a *Sepsis *specimen, as well as drawings by Ozerov [41] for a *Themira *specimen, but the most informative description thus far is by Eberhard and Huber [33] based on photos and drawings of several *Archisepsis *species. These authors document the presence of two spermathecae, a common oviduct, a vagina, a terminal sternite at the posterior end as well as an armored ovipositor wall. There are, however, disparities among the accounts. Eberhard and Kotrba identified a ventral receptacle, which is not mentioned by Ozerov. Similarly, Eberhard and Ozerov illustrated the presence of a large, dorsal vaginal sclerite/plate, which is not identified in Kotrba [40]. In addition, Eberhard's drawing indicates the presence of a smaller anterior sclerite and a ventral sac, which are not illustrated by the other two authors. These could be a result of species-specific differences, but at this point we just do not know enough about the internal female reproductive system of this family to be certain.

The Sepsidae are an acalyptrate family of flies of the Sciomyzoidea with approximately 320 described species in 37 genera. These flies are abundant worldwide and numerous species have broad distributions that span more than one continent [42]. Sepsid larvae and most adults are saprophagous i.e. closely associated with various decaying organic substrates. In recent years, sepsids have become models for testing sexual selection theories because they are strongly sexually dimorphic with respect to the male forelegs and terminalia and they also display elaborate courtship behavior [34,43-47]. The analysis presented here is part of a series of papers that uses a comparative approach to understanding the "creative powers" of sexual selection in diversifying morphology and behavior [47-50]. We provide the first comparative study of the internal female reproductive tract across Sepsidae (41 species representing 21 of the 37 described genera), using microscopic techniques that have been optimized to reveal even delicate structures such as the ventral receptacle.

Our study has three main aims. First, we investigate whether the internal female genitalia are species-specific in Sepsidae. For this purpose we include eight sister-species pairs that are very closely related based on morphological (male genitalia, male forelegs) and/or DNA sequence evidence. Second, we reconstruct the evolution of the female tract in Sepsidae by assembling a matrix based on characters describing the morphological variation in females and mapping it onto molecular [48,51] and morphological [52-54] phylogenetic hypotheses. We recently also published a comparative study on sepsid mating behavior [47] that documented rapid evolution and high levels of convergent evolution in behavioral characters that were comparable to fast evolving molecular characters such as the third positions in protein encoding mitochondrial genes. Our third aim is to assess whether the female reproductive system evolves similarly fast. We thus compare the number of character changes and quantify the level of convergence in our data set with the amount of change in mating behavior and the DNA barcoding gene used for species-identification [55,56]. We also identify and describe fast-evolving female structures that are likely candidates for future studies of post-copulatory sexual selection in Sepsidae.

## Methods

### 1) Testing the species-specificity of female genitalia in Sepsidae

#### Taxon sampling

We studied 41 species representing 21 of the 37 described genera (Table 1). To test the species-specificity of female structures, we included eight sister-species pairs that are closely related according to either male structures and/or DNA sequence evidence: (1) The common Holarctic *Themira biloba *and *T. putris *are morphologically so similar that they were considered one species until 1975 (Andersson, 1975); (2) *Meroplius fukuharai *was only described in 1984 because it is very similar to *M. minutus *[57]. The remaining sister pairs differ by <2% for the DNA barcoding gene *COI *(see discussion): (3) *Dicranosepsis emiliae *&*D. hamata*, (4) *Sepsis cynipsea *&*S. neocynipsea*, (5) *S. duplicata *&*S. secunda*, (6) *S. fulgens *&*S. orthocnemis*, (7) *S. punctum *&*S. monostigma*, and (8) *T. flavicoxa *&*T. lucida *[51].

**Table 1:** List of species included in study

Species	No. of specimens	Condition of specimens	Behavior data
Sepsidae			
*Allosepsis indica *(Wiedemann, 1824)	7	Freshly killed	Available
*Archisepsis scabra *(Loew, 1861)	1	Preserved in alcohol	-
*Australosepsis niveipennis *(Walker, 1860)	5	Freshly killed	Available
*Decachaetophora aeneipes *(de Meijere, 1913)	7	Freshly killed	Available
*Dicranosepsis crinita *(Duda, 1926)	5	Freshly killed	-
*Dicranosepsis distincta *Iwasa et Tewari, 1990	5	Freshly killed	-
*Dicranosepsis emiliae *(Ozerov, 1992)	5	Freshly killed	-
*Dicranosepsis hamata *(de Meijere, 1911)	5	Freshly killed	-
*Lasionemopoda hirsuta *(de Meijere, 1906)	4	Preserved in alcohol	-
*Meroplius fukuharai *(Iwasa, 1984)	5	Freshly killed	Available
*Meroplius minutus *(Wiedemann, 1830)	7	Freshly killed	-
*Mircosepsis armillata *(Melander & Spuler, 1917)	2	Preserved in alcohol	-
*Nemopoda nitidula *(Fallén, 1820)	5	Freshly killed	Available
*Ortalischema albitarse *Frey, 1925	5	Preserved in alcohol	-
*Orygma luctuosum *Meigen, 1830	7	Preserved in alcohol	Available
*Paleosepsis *sp.	2	Preserved in alcohol	-
*Parapalaeosepsis plebeia *(Meijere, 1906)	3	Preserved in alcohol	Available
*Paratoxopoda amonanae *Vanschuytbroeck, 1961	5	Preserved in alcohol	-
*Perochaeta dikowi *Ang et al. 2008	3	Preserved in alcohol	Available
*Platytoxopoda *spec.	1	Preserved in alcohol	-
*Saltella sphondylii *(Schrank, 1803)	4	Preserved in alcohol	-
*Sepsis cynipsea *(Linnaeus, 1758)	5	Freshly killed	Available
*Sepsis dissimilis *Brunetti, 1909	5	Freshly killed	Available
*Sepsis duplicata *Haliday, 1838	5	Freshly killed	-
*Sepsis flavimana *Meigen, 1826	6	Freshly killed	Available
*Sepsis fulgens *Meigen, 1826	5	Freshly killed	-
*Sepsis monostigma *Thomson, 1869	5	Freshly killed	-
*Sepsis neocynipsea *Melander & Spuler, 1917	5	Freshly killed	Available
*Sepsis orthocnemis *Frey, 1908	5	Freshly killed	-
*Sepsis punctum *(Fabricius, 1794)	5	Freshly killed	Available
*Sepsis secunda *Melander & Spuler, 1917	5	Freshly killed	Available
*Sepsis thoracica *(Robineau-Desvoidy, 1830)	5	Freshly killed	-
*Susanomira caucasica *Pont, 1987	2	Pinned	-
*Themira annulipes *(Meigen, 1826)	5	Freshly killed	Available
*Themira biloba *Andersson, 1975	5	Freshly killed	Available
*Themira flavicoxa *Melander & Spuler, 1917	5	Freshly killed	Available
*Themira lucida *(Staeger in Schixdte, 1844)	5	Freshly killed	Available
*Themira minor *(Haliday, 1833)	5	Freshly killed	Available
*Themira putris *(Linnaeus, 1758)	5	Freshly killed	Available
*Themira superba *(Haliday, 1833)	5	Freshly killed	Available
*Toxopoda *spec.	1	Preserved in alcohol	Available
Ropalomeridae			
*Willistoniella pleuropunctata *(Wiedemann, 1824)	2	Preserved in alcohol	-

List of 41 sepsid species and a ropalomerid outgroup, *Willistoniella pleuropunctata*. The table includes information on the specimen number and condition prior to dissection, and identifies the species for which data on mating behavior are available.

#### Morphological study

Our investigation of the female reproductive tract is primarily based on dissections of freshly killed and alcohol-preserved specimens. Only for *Susanomira caucasica *did we use pinned material (Table 1). Live flies were killed either by freezing or with a killing jar and dissected immediately in Ringer's solution under a Leica MZ16 microscope, while alcohol preserved and pinned specimens were macerated in KOH prior to dissection. Details on the external morphology of the female genitalia were not included in this study as there was not much change across the family. After dissection, we removed the internal reproductive tracts and mounted them, usually in a dorsoventral orientation, on glass slides in polyvinyl-lactophenol with an admixture of chlorazol black E. This medium progressively macerates the soft tissue while staining unsclerotized cuticular elements of the specimen blue. Dissections of fresh material yielded better results especially with respect to the membranous parts. Nonetheless, macerated specimens were sufficient for identifying the most important structures.

The slide preparations were studied under bright field and differential interference contrast (DIC) using a Zeiss Axioskop 2 equipped with a drawing tube as well as a Zeiss AxioCam digital camera. We prepared schematic drawings as well as photographic images at high magnifications for all the species. Descriptions of the internal reproductive tract are based on one to seven dissections per species (Table 1). The drawings focus on those structures that are ectodermal in origin and are lined with cuticle, thus omitting ovaries, lateral and common oviducts. The terminology of the morphological structures follows a recent glossary by Kotrba [58]. The term "sclerotized" refers to darkened cuticular structures that are not stained blue by chlorazol black E and appear brown under the bright field.

#### Sister-species comparisons

A character matrix was assembled for 41 sepsid species plus *Willistoniella pleuropunctata *(Ropalomeridae) as outgroup using MacClade 4.0 [59]. Nineteen morphological characters were defined based on the discontinuous variation of the female reproductive tract (Table 2). In order to test for species-specificity, we used the matrix to identify species that have identical character scores for the discontinuous variation. For these species pairs, we then assessed whether morphological structures with continuous variation across the family allowed for the differentiation of these species. In addition, using PAUP* [60], we calculated the uncorrected pairwise distances based on our female morphological data set as well as the distances for the *COI *barcoding gene [55] in order to compare the amount of character change across multiple data sets.

**Table 2:** Character matrix based on internal female reproductive structures

	0									1									
Species	1	2	3	4	5	6	7	8	9	0	1	2	3	4	5	6	7	8	9
*Allosepsis indica*	1	0	6	1	-	0	0	1	0	1	0	0	1	1	0	0	1	1	1
*Archisepsis scabra*	0	0	4	1	-	0	0	1	0	0	0	0	1	1	1	1	1	1	4
*Australosepsis niveipennis*	0	1	4	1	-	0	0	1	0	0	0	0	1	1	0	0	1	1	1
*Decachaetophora aeneipes*	0	4	4	2	4	-	0	1	0	0	1	0	0	0	0	0	1	1	7
*Dicranosepsis crinita*	0	0	3	1	-	1	0	1	0	0	0	0	0	1	1	0	1	1	5
*Dicranosepsis distincta*	0	0	4	1	-	1	0	1	0	0	0	0	0	1	1	0	1	1	5
*Dicranosepsis emiliae*	0	0	4	1	-	1	0	1	0	0	0	0	0	1	1	0	1	1	5
*Dicranosepsis hamata*	0	0	4	1	-	1	0	1	0	0	0	0	0	1	1	1	1	1	5
*Lasionemopoda hirsuta*	0	0	4	1	-	0	0	1	0	0	0	0	1	1	1	0	1	0	1
*Meroplius fukuharai*	0	0	5	1	-	0	0	1	0	0	0	0	0	2	1	0	1	1	5
*Meroplius minutus*	0	0	5	1	-	0	0	1	1	0	1	0	0	1	1	0	1	1	5
*Mircosepsis armillata*	0	0	4	1	-	0	0	1	0	0	0	0	0	1	1	1	1	1	4
*Nemopoda nitidula*	0	0	4	2	7	-	0	1	0	0	1	0	0	0	1	0	0	1	8
*Ortalischema albitarse*	0	2	0	2	5	-	0	0	-	-	0	0	0	0	1	1	0	0	1
*Orygma luctuosum*	0	0	-	-	-	-	0	0	-	-	0	0	0	0	1	1	0	0	1
*Paleosepsis spec.*	0	0	4	1	-	0	0	1	0	0	0	0	1	1	1	1	1	1	4
*Parapalaeosepsis plebeia*	0	1	3	1	-	0	0	1	0	2	0	0	1	1	0	1	1	1	4
*Paratoxopoda amonanae*	0	0	4	1	-	0	0	1	1	2	0	0	0	1	1	0	1	1	8
*Perochaeta dikowi*	0	0	3	1	-	0	0	1	0	0	0	0	1	1	1	0	1	0	6
*Platytoxopoda *spec.	0	3	-	1	-	0	1	1	1	0	0	0	0	1	0	1	1	1	0
*Saltella sphondylii*	0	0	4	2	3	-	0	1	0	0	0	0	0	1	0	1	1	1	1
*Sepsis cynipsea*	0	0	5	1	-	4	0	1	0	0	0	0	0	1	0	0	1	1	1
*Sepsis dissimilis*	0	0	3	1	-	0	0	1	0	0	0	0	0	1	0	0	1	1	3
*Sepsis duplicata*	0	0	3	1	-	3	0	1	0	0	0	0	0	1	1	0	1	1	3
*Sepsis flavimana*	0	0	4	1	-	6	0	1	0	0	0	0	0	1	1	0	1	1	3
*Sepsis fulgens*	0	0	2	1	-	4	0	1	0	0	1	0	0	1	1	0	1	1	1
*Sepsis monostigma*	0	0	4	1	-	2	0	1	0	0	1	0	0	1	1	0	1	1	1
*Sepsis neocynipsea*	0	0	5	1	-	3	0	1	0	0	0	0	0	1	0	0	1	1	1
*Sepsis orthocnemis*	0	0	3	1	-	4	0	1	0	0	1	0	0	1	1	0	1	1	1
*Sepsis punctum*	0	0	4	1	-	5	0	1	0	0	1	0	0	1	0	0	1	1	1
*Sepsis secunda*	0	0	3	1	-	3	0	1	0	0	0	0	0	1	1	0	1	1	3
*Sepsis thoracica*	0	0	5	1	-	3	0	1	0	0	0	0	0	1	1	0	1	1	1
*Susanomira caucasica*	0	0	6	2	8	-	0	1	0	0	0	0	0	0	1	1	1	1	2
*Themira annulipes*	0	0	2	2	6	-	0	1	0	0	0	0	0	0	1	0	1	0	1
*Themira biloba*	0	1	2	2	4	-	0	1	0	0	1	0	0	0	0	0	0	0	1
*Themira flavicoxa*	0	0	2	2	0	-	0	1	0	2	0	0	1	0	1	1	0	0	1
*Themira lucida*	0	0	1	2	0	-	0	1	0	2	0	0	1	0	1	1	0	0	1
*Themira minor*	0	0	1	2	1	-	0	1	0	2	0	0	0	0	1	1	0	0	1
*Themira putris*	0	0	3	2	2	-	0	1	0	0	1	0	0	0	1	0	0	0	1
*Themira superba*	0	0	2	2	0	-	0	1	0	2	0	0	1	0	1	0	0	0	1
*Toxopoda *spec.	0	3	-	1	-	0	1	1	1	0	0	1	0	1	0	1	1	1	0
*Willistoniella pleuropunctata*	0	0	-	0	-	0	0	0	-	-	0	0	0	0	1	0	-	-	-

Nineteen morphological characters scored across 41 sepsid species and the outgroup. Characters for: Ventral receptacle = 1-7; Dorsal sclerotization = 8-11; Spermathecae and spermathecal ducts = 12-16; Sternite VIII = 17; Ovipositor = 18 & 19.

### 2) Reconstructing the evolution of female genitalia

There is indication in sepsids that the phylogenetic signal from molecular sequences can conflict with that of morphological data [51]. We therefore mapped our female morphological characters onto two different phylogenetic hypotheses. Using PAUP* [60], we generated a molecular phylogeny based on DNA sequence data of 10 gene fragments from Ang et al.[48] and Su et al.[51] (87 sepsid species + 2 outgroups; heuristic search; 100 random stepwise additions; TBR branch swapping). A new analysis was needed to place the recently described *Perochaeta dikowi*, which is included in our study and whose DNA sequence data was added to Su et al.'s data using the concatenation software SequenceMatrix [61]. We also mapped the characters onto an earlier morphological phylogeny that was reconstructed based on egg, larval, and adult characters [52-54]. Our study of the female reproductive system has complete taxon overlap with the molecular phylogeny, while only 38 species are shared with the morphological tree; i.e., some species had to be deleted for the pairwise comparison with the morphological tree. The character mapping was performed in MacClade 4.0 [59] and we recorded the amount of change (tree length) and level of homoplasy as quantified by the consistency index (CI). Given that taxon number was kept constant in the pairwise comparisons, this avoids the problem of a negative correlation between CI and the number of terminals [62].

### 3) Quantifying the amount of change

To estimate the rate of change in the internal female reproductive characters, we compared our female morphological data set with other characters that are known to evolve fast such as the third positions of the mitochondrial DNA barcoding gene [55] and sepsid mating behavior [47]. We split the *COI *barcoding gene according to codon positions and obtained a character set containing only the non-constant third positions in order to ensure comparability to our morphological data, which also includes only non-constant characters. Due to incomplete taxon overlap between the behavior and female morphology data, we could only include the 22 species for which both data are available (Table 1). In MacClade 4.0 [59], we subsequently mapped all the different characters on the molecular phylogeny derived from the earlier-mentioned phylogenetic analysis. In order to assess the degree of homoplasy in our female reproductive characters, we mapped them onto the molecular phylogeny and calculated the number of observed steps over the minimum number of steps. This value quantifies the level of homoplasy and is the inverse of the consistency index. We repeated this for both the molecular and behavioral data set.

In addition, we split the morphological characters relating to the female reproductive tract according to different reproductive structures (e.g.: ventral receptacle, dorsal sclerite, spermathecae, etc.) and mapped them separately on the molecular tree in order to quantify the amount of change contributed by the individual structures.

## Results

### 1) Testing the species-specificity of female genitalia in Sepsidae

#### Morphological descriptions

##### *General description *Sepsidae

**Figure 1:**
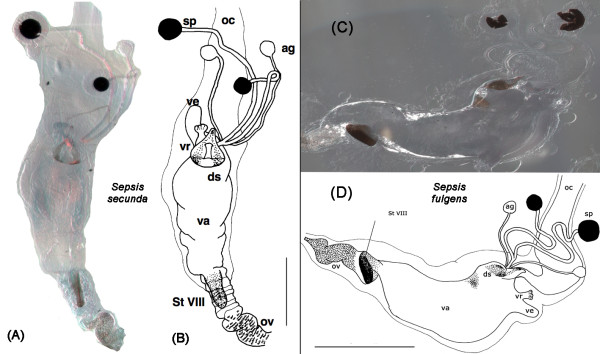
**Overview of female reproductive tract in *Sepsis secunda *Melander & Spuler 1917 [dorso-ventral orientation] and in *Sepsis fulgens *Meigen 1826 [lateral orientation]**. (A, C) Photograph of female tract taken under differential interference contrast (DIC); (B, D) Schematic illustration of female tract depicting the common oviduct (oc), vagina (va), two spermathecae (sp), the two accessory glands (ag), ventral evagination (ve), ventral receptacle (vr), dorsal sclerite (ds), sternite VIII (StVIII) and ovipositor (ov) [scale bar = 0.5 mm].

**Figure 2:**
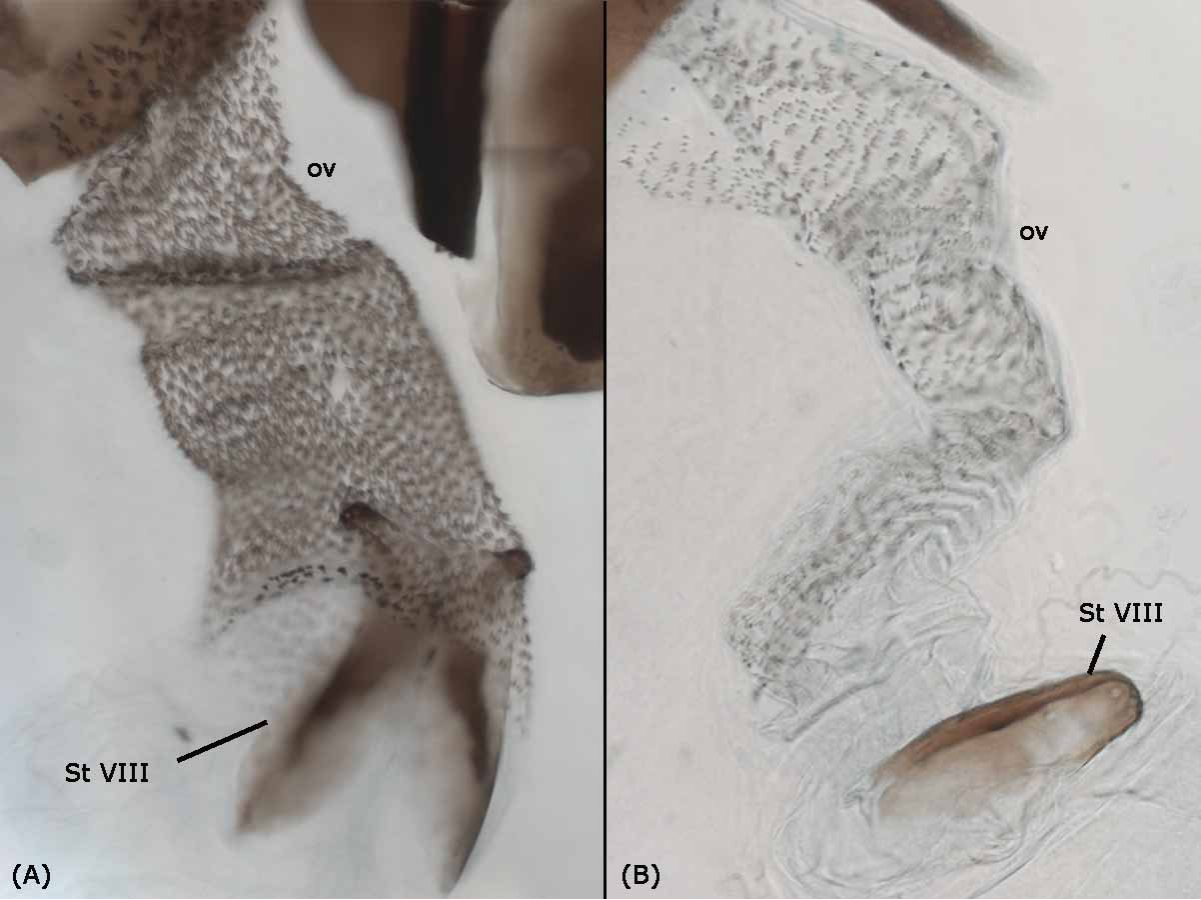
**Morphology of ovipositor (ov) and Sternite VII (StVIII)**. (A) Ovipositor wall is densely covered with large, single and evenly spaced spines, and posterior part of sternite VIII is clearly bifurcated in *Themira biloba*; (B) Ovipositor wall is lined with tiny spines in regular rows, and posterior part of sternite VIII is fused in *Dicranosepsis crinta*

**Figure 3:**
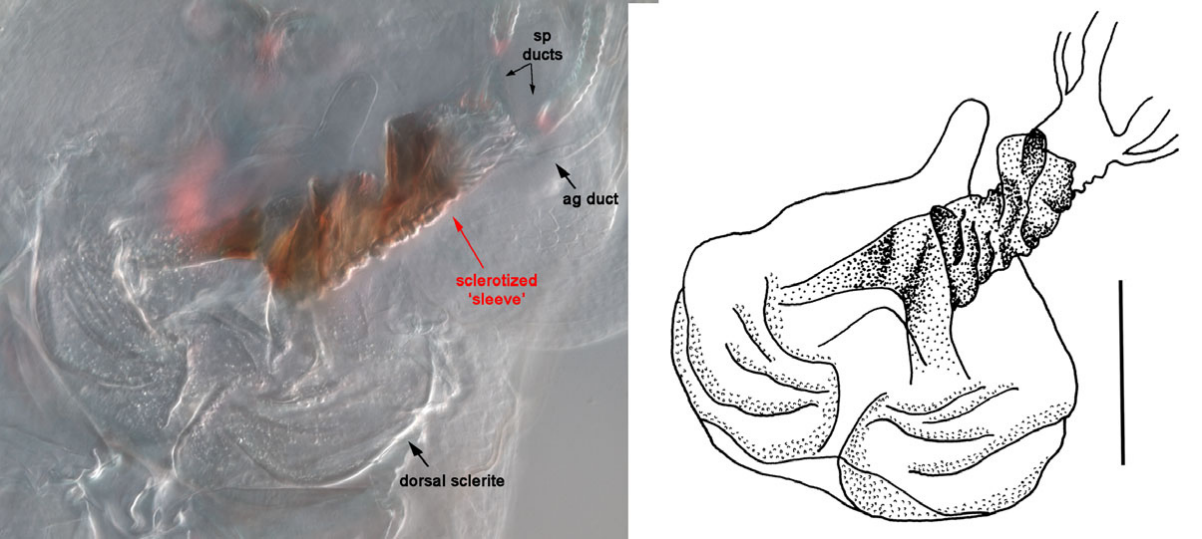
**Closeup of the sclerotized 'sleeve' in *Meroplius fukuharai***. Sclerotized sleeve-like pouch associated with the dorsal sclerite as well as the spermathecal and accessory gland duct openings in *Meroplius fukuharai *[scale bar = 0.1 mm].

**Figure 4:**
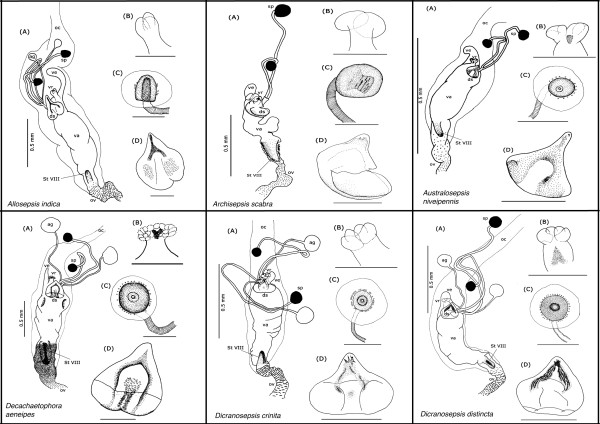
Schematic illustration of *Allosepsis indica, Archisepsis scabra, Australosepsis niveipennis, Decachaetophora aeneipes, Dicranosepsis crinita *and *D. distincta*

**Figure 5:**
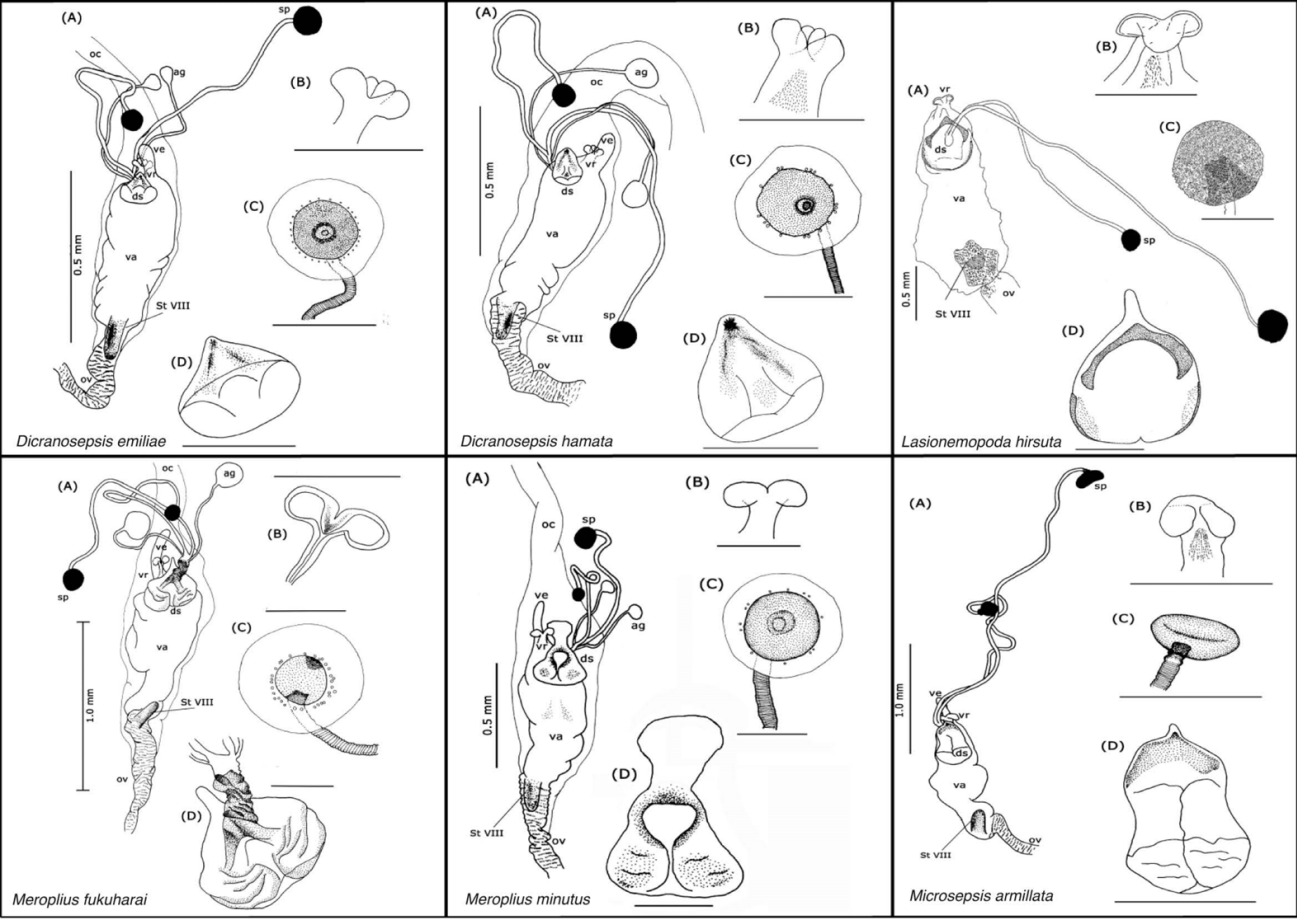
Schematic illustration of *Dicranosepsis emiliae, D. hamata, Lasionemopoda hirsuta, Meroplius fukuharai, M. minutus *and *Microsepsis armillata*

**Figure 6:**
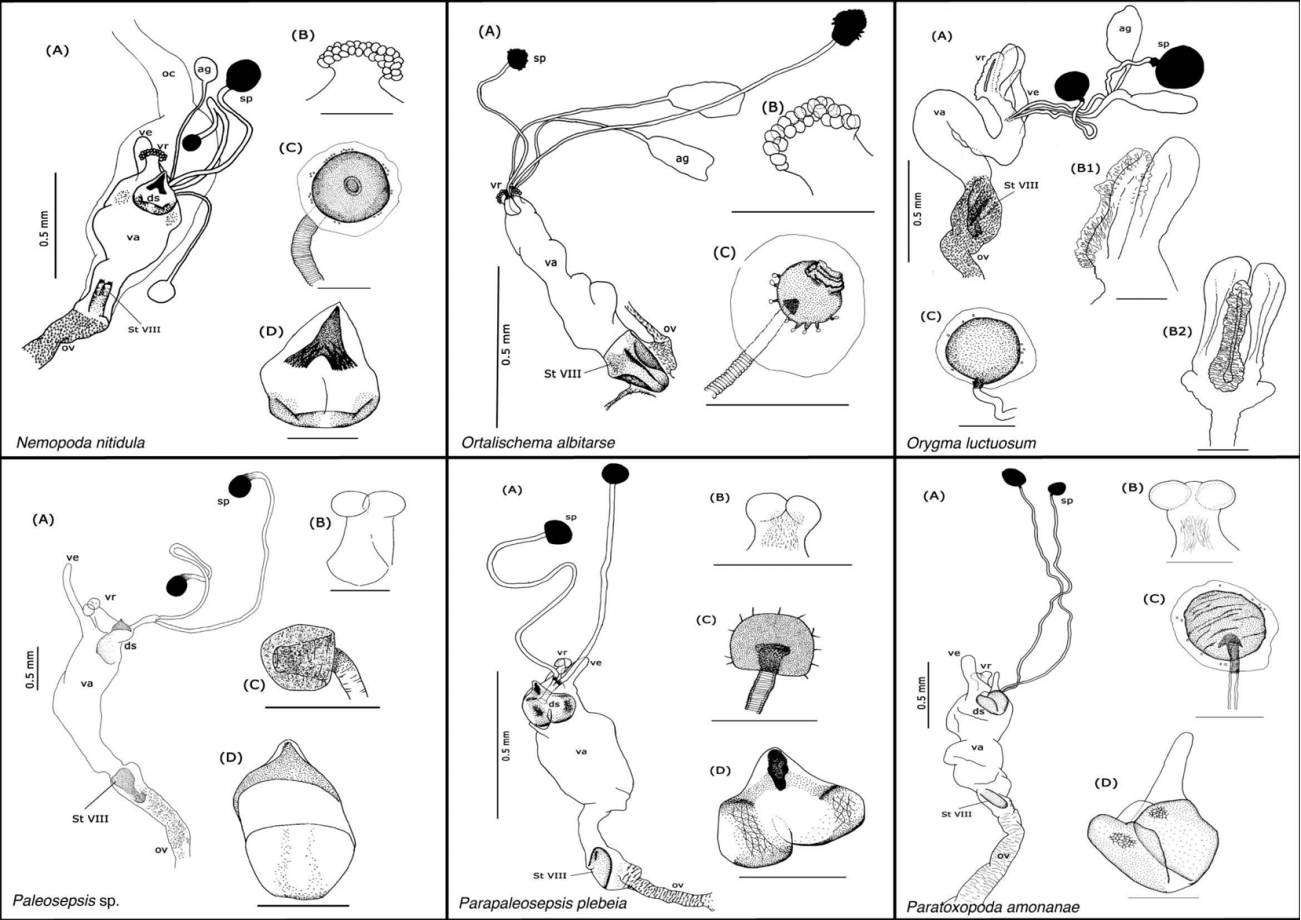
Schematic illustration of *Nemopoda nitidula, Ortalischema albitarse, Orygma luctuosum, Paleosepsis *sp., *Parapaleosepsis plebeia *and *Paratoxopoda amonanae.*

**Figure 7:**
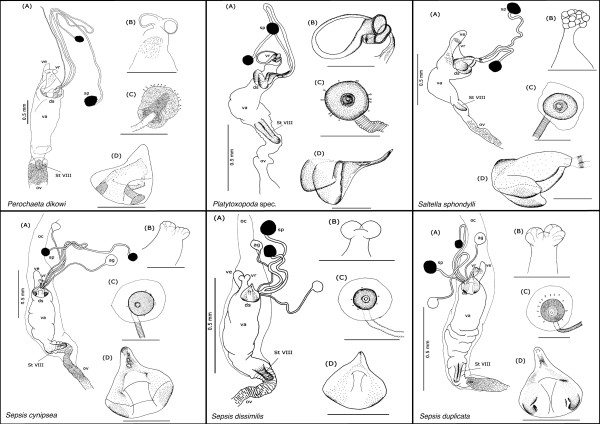
**Schematic illustration of *Perochaeta dikowi, Platytoxopoda *spec., *Saltella sphondylli, Sepsis cynipsea, S. dissimilis *and *S. duplicata***.

**Figure 8:**
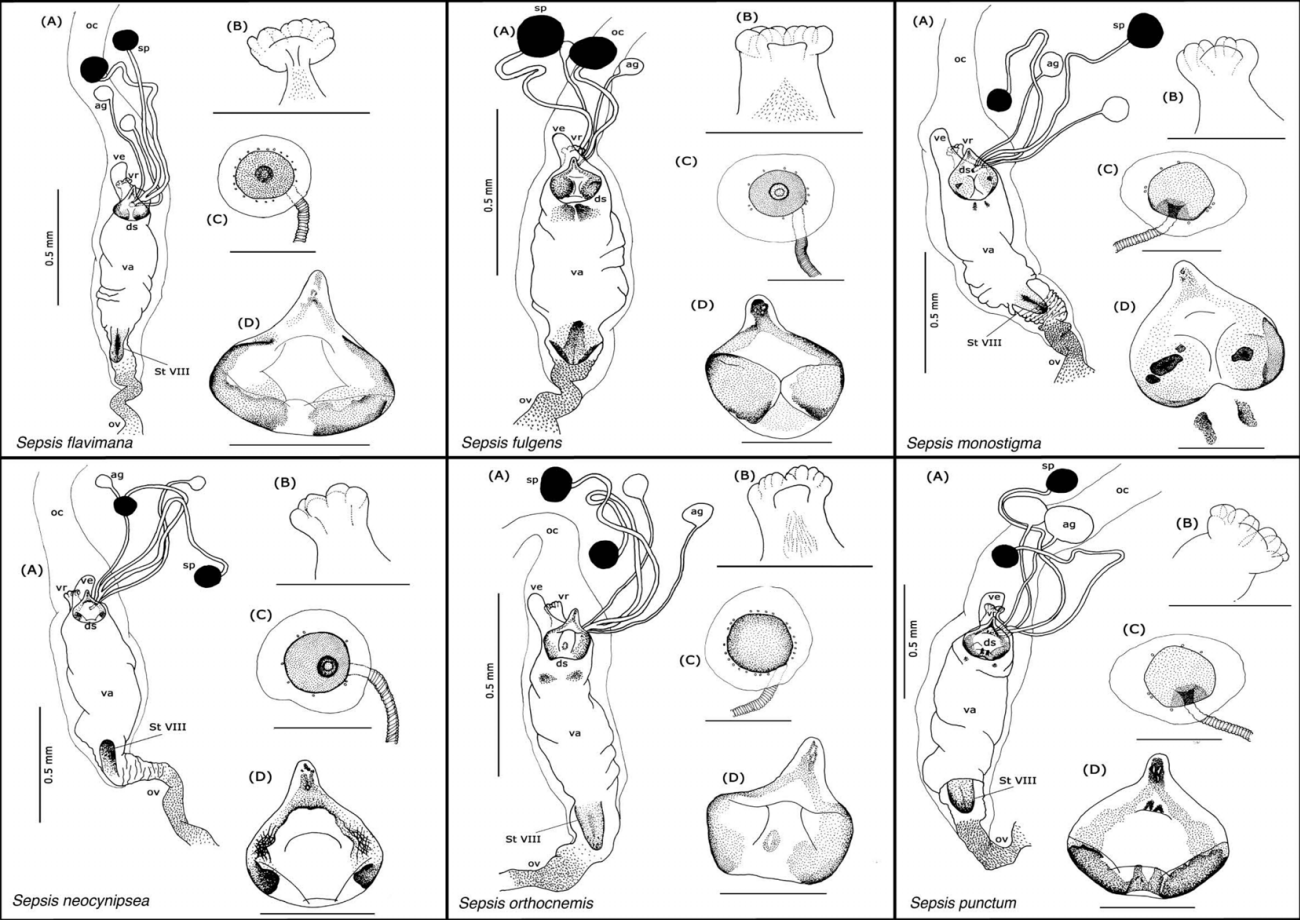
**Schematic illustration of *Sepsis flavimana, S. fulgens, S. monostigma, S. neocynipsea, S. orthocnemis *and *S. punctum***.

**Figure 9:**
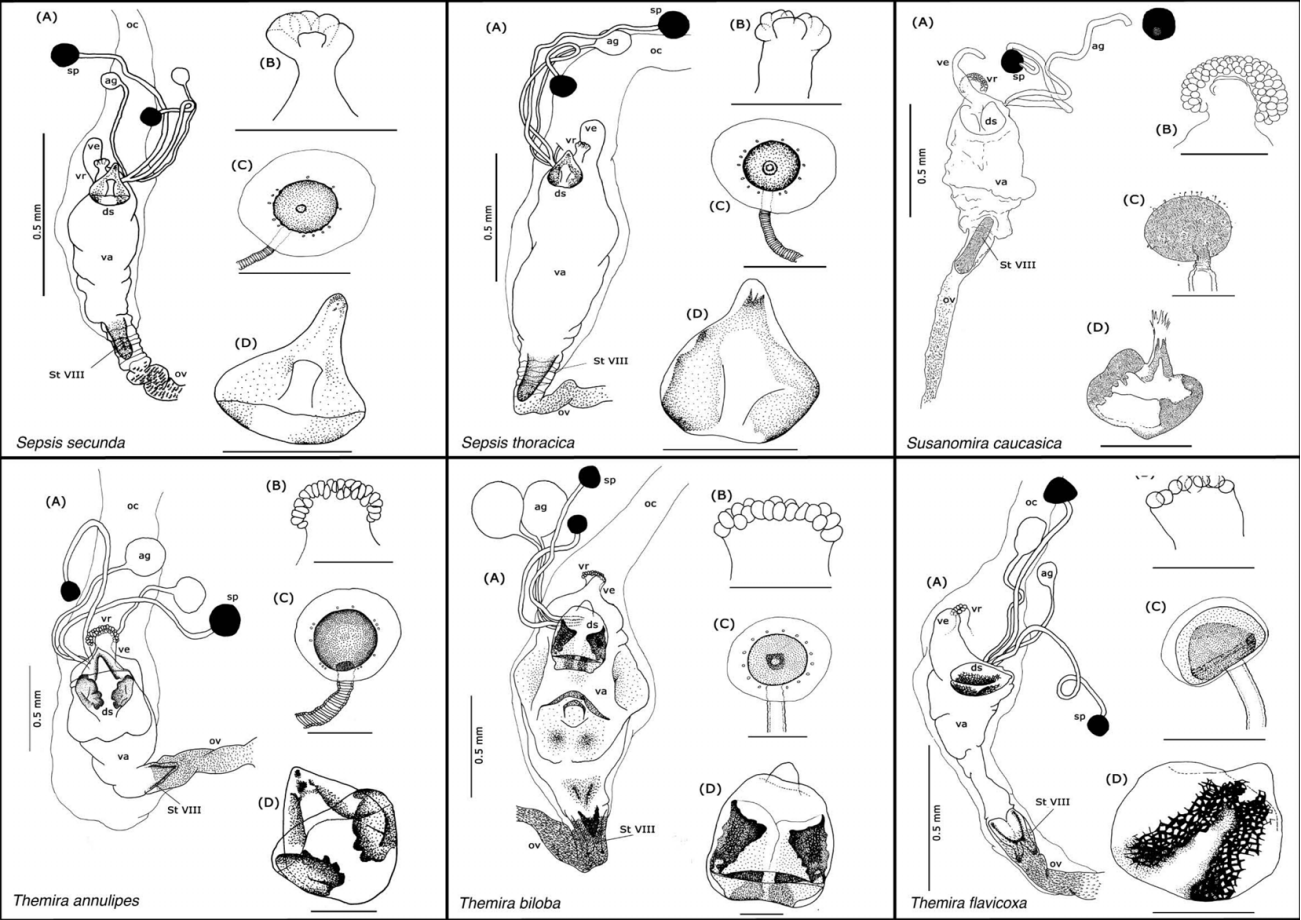
**Schematic illustration of *Sepsis secunda, S. thoracica, Susanomira caucasica, Themira annulipes, T. biloba *and *T. flavicoxa***.

**Figure 10:**
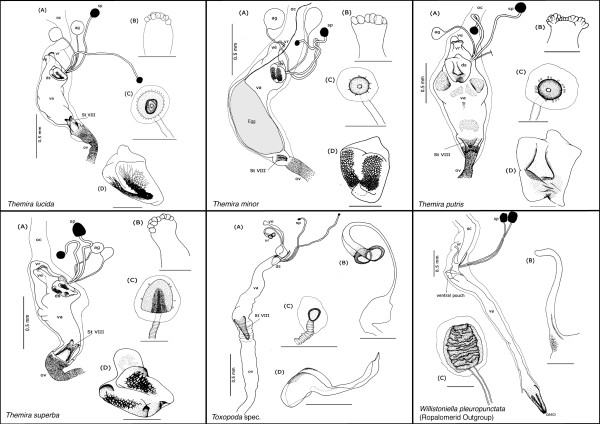
**Schematic illustration of *Themira lucida, T. minor, T. putris, T. superba, Toxopoda *spec. and outgroup *Willistoniella pluropunctata***.

Our description is restricted to the cuticular elements of the reproductive tract, which are: the tubular vagina (**va**), paired dorsal spermathecae (**sp**) and accessory glands (**ag**), the dorsal sclerite (**ds**; absent in *Orygma luctuosum *and *Ortalischema albitarse*), the ventral receptacle (**vr**), the ventral evagination (**ve**), the tubular inverted ovipositor (**ov**), and the internalized sternite VIII (**St VIII**). Please refer to the figures 1, 2, 3, 4, 5, 6, 7, 8, 9 and 10 when reading and evaluating the descriptive text.

The vagina is an elongate muscular tube lined internally with thin cuticle. It is anteriorly fused with the common oviduct (**oc**). The spermathecal ducts and the ducts of the accessory glands respectively open anterodorsally and posterolaterally into the vagina (Figure 1). This region of the vagina wall associated with the duct openings is, with the exception of *Ortalischema albitarse *and *Orygma luctuosum *(Figure 6), modified into a dorsal sclerite (**ds**), which can be very diverse in shape and degree of sclerotization. In some species, additional paired sclerotizations [e.g.: *Sepsis fulgens *(Figure 1) and *Themira biloba *(Figure 9)] occur in the posterior dorsal vaginal wall. Directly opposite to the spermathecal duct openings, the anteroventral portion of the vagina gives rise to the ventral receptacle and, directly posterior to this, the ventral evagination. When the vagina is empty, its wall forms numerous folds. This allows the vagina to become greatly extended when containing a spermatophore after copulation or, in the case of viviparous species, a developing egg or larva [35]. In our dissections we observed the vagina to contain spermatophores in some species [e.g: *Allosepsis indica, Australosepsis niveipennis*, *Sepsis dissimilis *and *S. thoracica*] and a single developing egg in *Themira minor *(Figure 10).

The vagina tapers and opens into the likewise tubular ovipositor, which, together with sternite VIII, is invaginated from the body surface between sternite VII and the terminalia. In some species, the apical portion of sternite VIII is clearly bifurcated, while in others it is fused. The ovipositor wall is, with the exception of *Toxopoda *and *Platytoxopoda*, internally ornamented to a variable degree with spines or tiny denticles that may be arranged as singles, clusters or rows (Figure 2).

All sepsids have two strongly sclerotized spermathacae that are usually round, although ovoid, mushroom- and barrel-shaped forms also occur. They are at times highly telescoped [e.g: *Allosepsis indica *(Figure 4) and *Themira superba *(Figure 10)]. The spermathecal capsules can be of equal or, more often, of distinctly unequal size. In one species, *Toxopoda sp.*, we found the spermathecal capsules to be almost entirely reduced (Figure 10). Moreover, in some species their wall is ornamented with transverse wrinkles or spines [e.g.: *Paratoxopoda amonanae *(Figure 6) and *Platytoxopoda *(Figure 7) respectively]. The latter are usually connected to cuticular end apparatuses of gland cells in the surrounding glandular tissue. In *Ortalischema albitarse*, the spermathecae have a particularly elaborate shape with a belt of spines around their perimeter and an apical spiny crown (Figure 6).

The spermathecal ducts can likewise be either equal or unequal in length. They can (i) open near to each other but separately into the dorsal vaginal wall [e.g.: *Themira biloba *(Figure 9)], or (ii) open into a common pouch of the vagina wall, sometimes together with the accessory gland ducts [e.g.: *Archisepsis scabra *(Figure 4)]. This pouch can be of considerable length. In one species, *Meroplius fukuhari*, it forms a heavily sclerotized 'sleeve' that is associated with the dorsal sclerite (Figure 3). In a few other species, the bases or the apical portions of the spermathecal ducts themselves are weakly sclerotized [e.g.: *Parapaleosepsis plebeia *(Figure 6) and *Saltella sphondylii *(Figure 7)].

The accessory glands are lined by delicate cuticle, which is surrounded by a glandular epithelium. Their size varies across species and they are at times even larger than the spermathecae [e.g.: *Dicranosepsis crinita *(Figure 4) and *Themira minor *(Figure 10)]. The membranous accessory gland ducts are predominantly equal in length and shorter than the spermathecal ducts. Because of their very delicate nature they are often lost in dissections, especially after maceration, which is why they are distorted or lacking in some of our illustrations.

**Figure 11:**
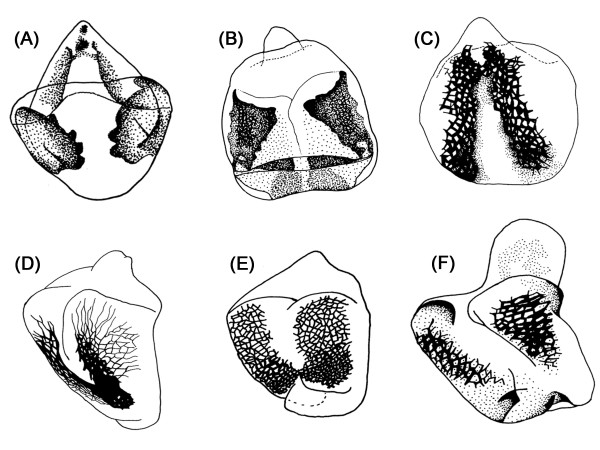
**Diversity of dorsal sclerite morphology within the genus *Themira *Robineau-Desvoidy 1830**. (A) *Themira annulipes*; (B) *T. biloba*; (C) *T. flavicoxa*; (D) *T. lucida*; (E) *T. minor*; (F) *T. superba*

In all species but *Ortalischema albitarse *and *Orygma luctuosum*, the openings of spermathecal and accessory gland ducts are framed by the dorsal sclerite, whose shape most usually resembles that of an inverted heart. It varies greatly across the family and even between closely related species within a genus (Figure 11). The difference in shape as well as in the degree and pattern of sclerotization is often difficult to describe or code as characters with discrete states. However there are some distinctive features, such as the presence of a distinct honeycomb surface ornamentation at the basal cheeks of the sclerite and the presence of a long process at its anterior part [e.g.: *Paratoxopoda amonanae *(Figure 6)].

**Figure 12:**
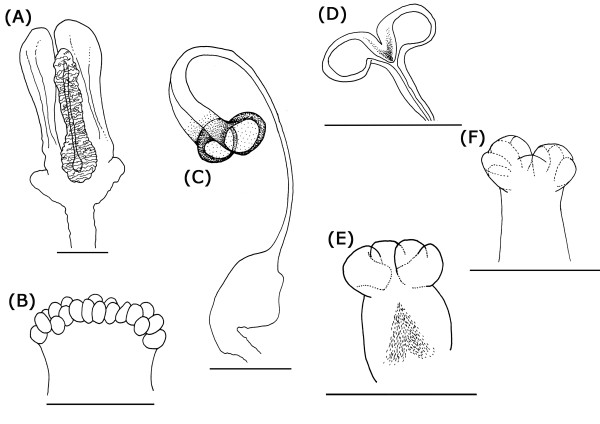
**Diversity of ventral receptacle (VR) morphology across Sepsidae**. (A) Autapomorphic VR in *Orygma luctuosum*; (B) Multi-chambered VR in *Themira biloba*; (C) Bi-lobed VR with a stalk-like extension of the base in *Toxopoda*; (D) Bi-lobed VR in *Meroplius fukuharai*l; (E) Bi-lobed VR with two secondary subdivisions in *Dicranosepsis distincta*; (F) Bi-lobed VR with multiple secondary subdivisions in *Sepsis duplicata *[scale bar = 0.1 mm].

The ventral receptacle arises from the anteroventral portion of the vagina and extends anteriorly along the basal part of the common oviduct. It is also very variable, albeit mostly between genera (Figure 12). In *Orygma*, this organ is very large and of unique shape compared to all other species. It has an elongate central rod that is quite massive and flanked by thin-walled lateral chambers (Figure 6). Due to its significant morphological difference, this structure is difficult to homologize with structures in the other remaining species. The ventral receptacle in other sepsids, can be bi-lobed with or without additional internal subdivisions; or it can be multi-chambered with 10 to 80 separate roundish chambers. The interpretation of the multi-chambered ventral receptacle condition in *Decachaetophora aeneipes *was somewhat difficult, because in all seven studied specimens this structure appeared somewhat disintegrated, although delicate chambers and a diffuse apical brown spot were discernible (Figure 4). In some species, the ventral receptacle has a more or less distinct sclerotized plate apically, and in one species, *Allosepsis indica*, this apical portion is enlarged to almost the size of an additional chamber (Figure 4). In the clade *Paratoxopoda *+ *Toxopoda *the ventral receptacle is completely sclerotized and has a modified stalked base (Figure 6 &7 respectively).

The ventral evagination inserts posterior of the ventral receptacle. From there it extends anteroventrally past the ventral receptacle. It varies in size, sometimes projecting further than the ventral receptacle, in other species not even reaching the length of that organ [e.g.: *Susanomira caucasica *(Figure 9) and *Themira lucida *(Figure 10) respectively]. This structure has been termed 'ventral sac' by Eberhard and Huber [33].

##### *Description of outgroup species *Willistoniella pleuropunctata *(Ropalomeridae)*

The cuticular elements include a tubular vagina, paired, elongate spermathecae and accessory glands. However, unlike in sepsids, the ventral receptacle is tubular and a dorsal sclerite is absent. Also, there is a small ventral pouch posterior to the ventral receptacle with unclear homology. In addition, instead of an inverted ovipositor, the vagina opens posteriorly into a fused sheath formed by segment VIII and contains the cerci (Figure 10).

#### Sister species comparisons

Based on our dissections of 41 sepsid species and the ropalomerid outgroup *Willistoniella pleuropunctata*, we defined 19 morphological characters describing the internal female reproductive tract and scored them across all taxa (Table 2; 11 binary, 8 multistate characters; 3 ordered, 16 unordered). The detailed character descriptions are provided as supplementary data (Additional file 1). Of the 42 species included in this study, only two pairs of taxa, *Dicranosepsis distincta *&*D. emiliae *(non-sister taxa) and *Sepsis duplicata *&*S. secunda *(sister taxa) have identical character codes for the female reproductive tract. All other species differ in at least one character.

For the eight sister-species pairs, we quantified the amount of changes separating them based on uncorrected pairwise distances (PD) derived from our female data set and the variable third positions for the *COI *barcoding gene. They are: (i) *Dicranosepsis emiliae - D. hamata *[PD_FEM _= 5.56%, PD_*COI *_= 1.49%]; (ii) *Meroplius fukuharai - M. minutus *[PD_FEM _= 16.7%, PD_*COI *_= 13.4%]; (iii) *Sepsis duplicata - S. secunda *[PD_FEM _= 0%, PD_*COI *_= 1.97%]; (iv) *S. cynipsea - S. neocynipsea *[PD_FEM _= 5.56%, PD_*COI *_= 1.34%]; (v) *S. fulgens - S. orthocnemis *[PD_FEM _= 5.56%, PD_*COI *_= 0.76%]; (vi) *S. monostigma - S. punctum *[PD_FEM _= 22.2%, PD_*COI *_= 3.25%]; (vii) *Themira biloba - T. putris *[PD_FEM _= 27.8%, PD_*COI *_= 9.37%]; (viii) *T. flavicoxa - T. lucida *[PD_FEM _= 5.56%, PD_*COI *_= 0.69%] (Figure 9). Seven of these pairs exhibit greater difference in the female characters than the *COI *barcode (χ^2 ^test, 1 df; p = 0.034).

### 2) Reconstructing the evolution of female genitalia

**Figure 13:**
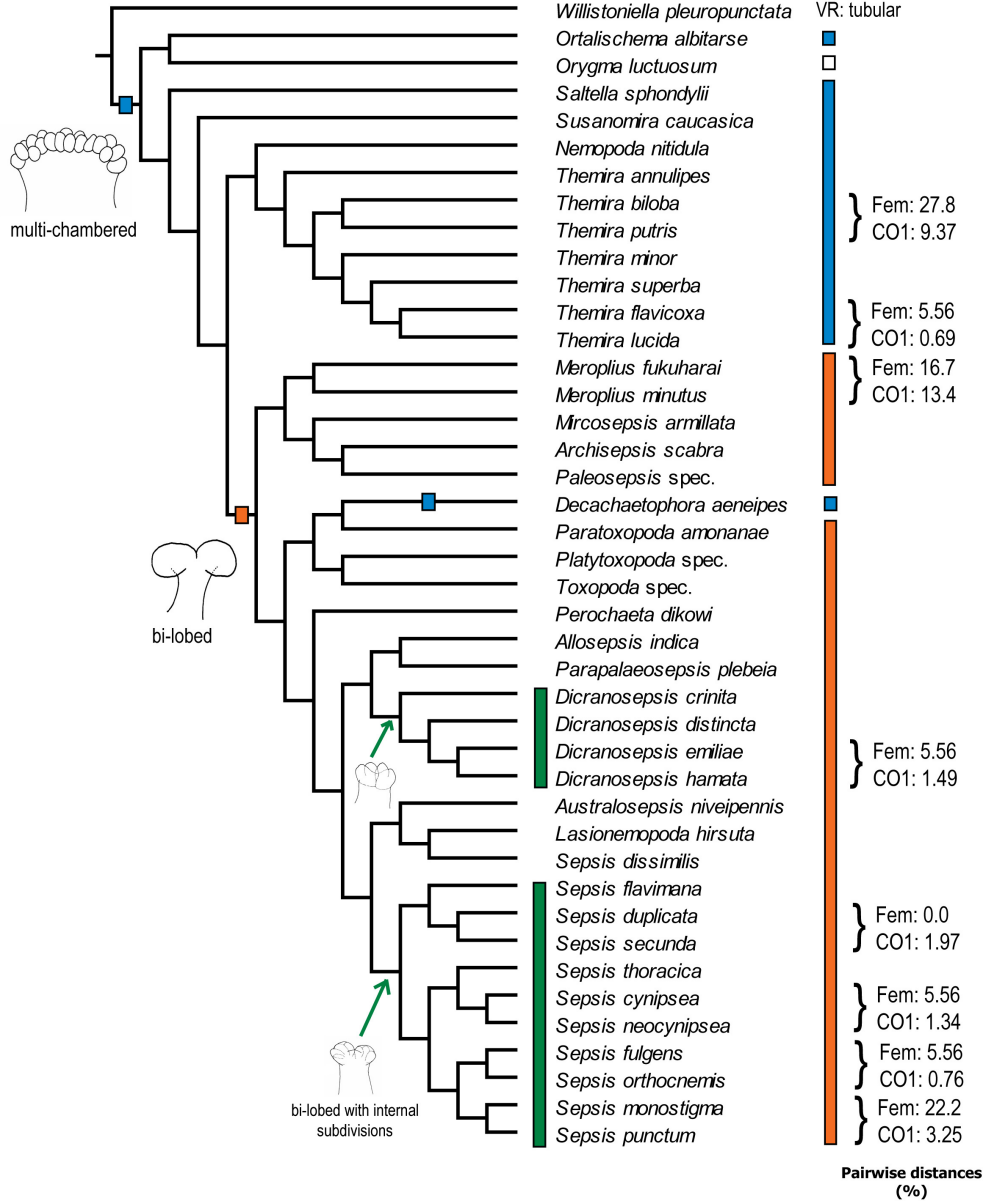
**Evolution of the multi-chambered and bi-lobed ventral receptacle in Sepsidae**. Blue hatchet/bars represent the multi-chambered state of ventral receptacle in sepsids and the orange hatchet/bars represent the bi-lobed state of ventral receptacle. The white bar represents the autapomorphic ventral receptacle of *Orygma luctuosum*. The green arrows/bars represent the separate origins of secondary internal subdivisions in the ventral receptacle of the *Dicranosepsis *and *Sepsis *groups.

Our heuristic search yielded three most parsimonious trees (19939 steps), and the strict consensus tree was pruned to include only the 42 species covered in this study. Given that the differences between the three equally parsimonious trees affect species that were not included here, the tree for our 42 species is fully dichotomous. We refer to this tree as the molecular phylogeny from this point forth (Figure 13). Tracing our morphological characters on the molecular phylogeny with 42 taxa required 120 steps (CI_MOL _= 0.41). For the comparison with the morphology-based phylogeny, we had to exclude taxa not included in the morphology-based hypotheses [52-54]. After culling these, our tree included 38 taxa and the female internal reproductive characters require five more steps than on the molecular tree. Overall, we thus found that the evolution of the female internal reproductive characters is more in line with the recent molecular phylogeny (113 steps; CI_MOLcul _= 0.42) than the earlier morphology-based hypothesis (125 steps; CI_MORPH _= 0.38). Hence, we decided to use the molecular phylogeny for the subsequent discussion of character evolution (Figure 13).

### 3) Quantifying the amount of change

The ventral receptacle morphology contributed seven of the 19 characters and accounted for 61 of the 120 steps. The remaining 12 characters were based on the dorsal sclerite (4 characters; 16 steps), ovipositor (2 characters; 13 steps), sternite VIII (1 character; 3 steps), spermathecae (3 characters; 15 steps) and spermathecal ducts (2 characters; 12 steps). We also quantified the level of homoplasy by computing the number of observed steps over the minimum number of steps for the characters (the inverse of the CI). Morphological characters related to the female reproductive tract have lower levels of homoplasy (Female: Obs./Min = 1.84) compared to non-constant third positions of the DNA barcoding gene (DNA: Obs./Min = 2.77) and the behavioral data (Behavior: Obs./Min = 2.21). (Additional file 2).

## Discussion

The morphology of female sperm storage organs could play an important role in post-copulatory sexual selection, influencing where the sperm is stored, how it is dispensed and whether it can be displaced. In sepsids, the main sperm storage organs are the two spermathecae and the ventral receptacle. The spermathecae are not greatly modified across the family, although there are differences in shape and size between species. Accordingly, the spermatheacae only contribute three of the 19 characters in our matrix, which account for 12.5% of the total change observed. The spermathecal ducts and ovipositor contribute even fewer changes (10% and 10.8% respectively), while the sternite VIII accounts for the least amount of change (2.5%). However, there are two features of the female reproductive tract that are evolving at a fast rate: the dorsal sclerite, which could be interacting with the male phallus and the ventral receptacle, which has been proposed as the likely the site of fertilization in several dipterans [63,64]. We believe that both structures are potential targets of post-copulatory sexual selection in Sepsidae.

### The species-specificity of the female reproductive system in Sepsidae

**Figure 14:**
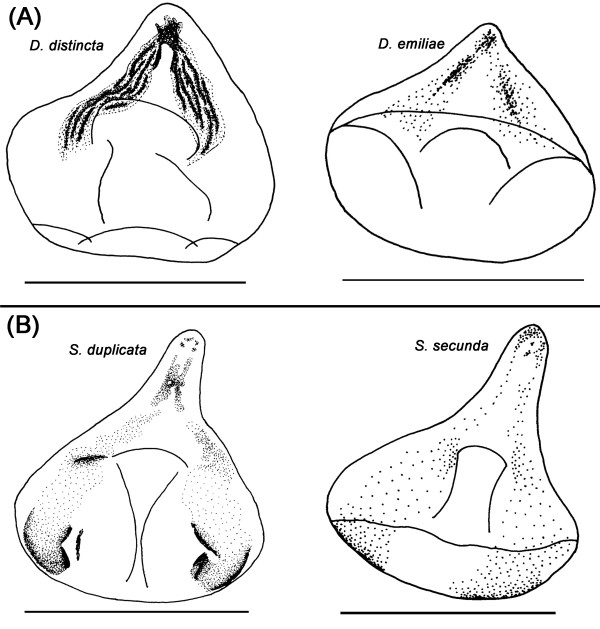
**Differences in pattern of sclerotization on dorsal sclerite among species pairs with identical character coding**. (A) Pattern of sclerotization on dorsal sclerite is different between non-sister taxa *Dicranosepsis distincta *and *D. emiliae*; (B) Sister taxa *Sepsis duplicata *and *S. secunda *also differ with regard to pattern of sclerotization on dorsal sclerite [scale bar = 0.1 mm].

With the exception of two pairs of species that are identical at the character matrix level, every other species has a unique combination of morphological characters (Table 2) and even the "identical" species differ with regard to morphological differences in characters that have continuous variation across the family. For both sister pairs [*Dicranosepsis distincta *&*D. emiliae *(Figure 14A) and *Sepsis duplicata *&*S. secunda *(Figure 14B)] the patterns of sclerotization on the dorsal sclerite differ. Although these differences are difficult to describe or code as characters with discrete states, they are nevertheless sufficiently distinct to allow for the unambiguous identification of the species. We thus find that closely related sepsid species do differ with respect to the female genitalia. In fact, based on the comparisons of the eight pairs of close relatives, all but *S. duplicata *and *S. secunda*, had higher pairwise distances in the female data than the *COI *barcode (χ^2 ^test, 1 df; p = 0.034).

But did these differences in the female reproductive tract evolve quickly? In the absence of a good fossil record for Sepsidae, we can only use molecular data as a point of comparison. Most species of Diptera differ by 3-5% for the *COI *barcode [56,65]. Several of the closely related species-pairs that we used in our study have much lower pairwise distances: *D. emiliae *and *D. hamata *(1.49%), *S. cynipsea *and *S. neocynipsea *(1.34%), *S. fulgens *and *S. orthocnemis *(0.76%), *Themira flavicoxa *and *T. lucida *(0.69%). These low genetic distances imply that the morphological differences in the female reproductive structures must have arisen very fast. There are many studies that focus on the rapid divergence and species-specificity of male structures and emphasize the lack of stark differentiation in female structures [6,8,9,15,66,67]. Our study indicates that, the internal female genitalia in Sepsidae are not only diverse but also evolve fast and are as diagnostic for species as male genitalia and forelegs.

### Fast-evolving female structures and their potential role in post-copulatory sexual selection

#### The dorsal sclerite

The dorsal sclerite evolves rapidly across the sepsid family, representing four characters in the matrix and 13.4% of all changes observed. This structure is variable even within a genus (Figure 11) and between closely related species. In Ozerov's schematic drawing of a *Themira *sp., he indicated that the female reproductive tract had a 'vaginal plate', and we believe he was referring to the dorsal sclerite [41]. However, he did not explain the functional significance of this structure. Eberhard and Huber [33] studied the copulation and sperm transfer in male *Archisepsis *and gave a detailed description as to how the male intromittent organ interacts with the female reproductive tract. They state that in addition to spiny erectable processes that anchored to the vaginal wall, the male aedaegus also had a surface densely covered with large, stiff bristles that can be pressed against the surface of a large 'vaginal sclerite' (i.e. the dorsal sclerite) [33]. Given this description, one possible reason for the species-specific diversity in the dorsal sclerite in females, is that it has co-evolved with the species-specific male intromittent organ with which it interacts.

Sexual conflict results from the asymmetries in evolutionary interests between males and females, particularly, in relation to the control over reproduction. Both sexes accumulate traits in the form of a coevolutionary sexual arms race, with males persisting to achieve copulation using 'weapons' and females resisting their attempts with 'defenses' [68,69]. Hence, both sexes are being pulled in opposing directions resulting in the exacerbation of these antagonistic adaptations [70,71]. In sepsis, recent artificial selection experiments in *Sepsis cynipsea *indicate that with increasing population densities and a polyandrous system, the harm inflicted by male persistence behaviors can result in the evolution of female resistance behaviors [45,72,73]. Hence, it is possible that the highly diverse dorsal sclerites documented in this study arose as a female response or defense to prevent and/or reduce harm caused by the heavily armored male genitalia (i.e. coevolutionary morphological arms race).

Another possible explanation is that the dorsal sclerites act as structures that detect male genital stimulation (i.e. female choice) [74]. In some tsetse flies, the males have been documented to stimulate the female internally with their aedaegus during copulation, prior to insemination [18,33-35]. In fact, in certain neotropical sepsids, males have been observed to repeatedly stimulate the females externally using their highly modified claspers to squeeze them in a rhythmic repertoire [44]. It is thus likely that the male intromittent organs are rubbing against the dorsal scleroses and serve as courtship signals to influence post-copulatory female choice. Unfortunately, the phallus of sepsid males are poorly studied because most species can be identified based on foreleg and claspers morphology. However, future studies should test whether male intromittent organs are also species-specific and how they interact with the female internal genitalia *during *copulation.

#### The ventral receptacle

The ventral receptacle displays a large amount of morphological variation across the Sepsidae, especially between genera (Figure 12). It contributes seven of the 19 female diagnostic characters and accounts for more than half of the overall evolutionary change observed (50.8%). From a multi-chambered state in the more basal taxa like *Susanomira caucasica, Saltella sphondylii *and *Ortalischema albitarse*, the number of chambers in the ventral receptacle decreases drastically across Sepsidae. This is particularly evident within the *Themira *clade where the number of chambers decreases from 27 to 10. In *Dicranosepsis *and *Sepsis*, the ventral receptacle appears multi-chambered as well (Figure 4,5 and 7-10 respectively). However, here the chambers are not clearly defined separate units. Instead, the two primary compartments are secondarily subdivided. The ventral receptacle in these is also clearly bi-lobed unlike the ones of *Saltella*, *Susanomira *or *Themira*. Based on the position of *Dicranosepsis *and *Sepsis *on the phylogenetic tree as well as the morphology of the internal subdivisions, we suggest that the multi-chambered ventral receptacle evolved twice (indicated by arrows in Figure 12).

The ventral receptacle in *Orygma luctuosum *is particularly interesting (Figure 6). Based on dissections of other acalyptrate flies (Kotrba unpublished), we believe the elongate central rod flanked by thin-walled lateral chambers of this species to be autapomorphic. Su et al.[51] discuss conflict between morphological and molecular data in placing *Ortalischema albitarse*, with molecular data placing it as sister species to *Orygma *and morphological data placing *Orygma *as sister to all remaining sepsids [54]. Given the unique ventral receptacle condition in *Orygma *and considering the multi-chambered ventral receptacle of *Ortalischema *(Figure 6; similar to others like *Themira *and *Saltella*), our data are in line with either hypothesis and cannot be used to resolve the position of *Ortalischema*.

Another remarkable ventral receptacle structure is observed in *Platytoxopoda *and *Toxopoda*. The base of the receptacle in these species extends as a stalk-like tube that terminates in two strongly sclerotized chambers (Figure 6 &10). These are absent in other sepsids. Interestingly, while the spermathecae of *Platytoxopoda *are of normal size, the spermathecae in *Toxopoda *are drastically reduced in size. Given these dramatic differences in the female reproductive tract, we suggest that these sister groups would be ideal for future comparative studies investigating the relative importance of the ventral receptacle versus the spermatheacae in sperm storage and fertilization. For example, some studies indicate that the evolution of female sperm storage organs drive the evolution of male sperm and sperm storage organs [22,26,75]. Hence, it would be interesting to test whether the length of the long, stalked ventral receptacle in *Platytoxopoda *and *Toxopoda *females is correlated with sperm length in the males of these species.

Considering the morphological diversity of the ventral receptacle in sepsids, the question arises as to its role in post-copulatory sexual selection. Some recent studies indicate that females are capable of biasing paternity by several means. For instance, females of the yellow dung fly *Scathophaga stercoraria *influence the traffic of sperm stored in the spermathecae to the point of fertilization [17,29,76] while in certain drosophilid groups females actively eject or 'dump' sperm [67]. The specific location where fertilization takes place is still unknown for most taxa and some authors speculate that the spermathecae provide spermatozoa directly to the eggs [77,78]. Yet, a study in *S. stercoraria*, documented that when the egg was in the vagina, the micropyle was oriented ventrally, away from the spermathecal duct openings which open dorsally into the vaginal wall [17]. Indeed, in many acalyptrate Diptera, the ventral receptacle is believed to be the more likely site of fertilization and some studies document the presence of spermatozoa stored in the ventral receptacle just prior to fertilization [28,79]. For instance, in ovipositing females of the Mediterranean fruit fly, *Ceratitis capitata*, the ventral receptacle was the first sperm storage organ to deplete, implying that it was the site where fertilization occurred [63], while in the Queensland fruit fly, *Bactrocera tryoni*, the spermathecae serve to replenish the ventral receptacle where a small number of sperm is kept for fertilizations [64].

Given that the ventral receptacle is the likely fertilization site in acalyptrate flies [80,81], finding great diversity in morphological structure in 41 species of Sepsidae is important. In other dipteran families like the Opomyzidae and the Tephritidae, the ventral receptacle is also multi-chambered and each chamber has been observed to house one to four spermatozoa [19,82]. In fact, in Drosophilidae, the length of the tubular ventral receptacle (also called the seminal receptacle) in females was suggested to drive the evolution of male sperm length [75]. The general reduction in ventral receptacle chamber numbers observed in sepsids prompts future work on the possible coevolution between the number and length of stored spermatozoa and the number and volume of chambers in the ventral receptacle.

### Convergent evolution of female reproductive structures in Sepsidae

We have documented that the female genitalia are diverse across the Sepsidae, but just how fast and homoplasious are these changes? To answer these questions, we compared our female data with other character systems such as the non-constant third positions of the mitochondrial *COI *barcoding gene [55] and sepsid mating behavior [47]. By calculating the proportion of observed changes to minimum changes, we derived convergence estimates that indicate that the female morphological characters are evolving at only approximately 2/3 the rate of molecular data (Fem: Obs./Min = 1.84; CI = 0.54). They are also not as homoplasious as the third positions (*COI*: Obs./Min = 2.77; CI = 0.36) or the mating behavior (Behav: Obs./Min = 2.21; CI = 0.45) (Additional file 2). However, it is important to remember that third positions of mitochondrial protein-encoding genes evolve particularly fast and so overall, the sepsid female reproductive tract can be considered a fast-evolving structure.

## Conclusions

We document that in sepsid flies the internal female genitalia are diverse and are evolving at a much faster rate than conventionally assumed. This applies in particular to the dorsal sclerite and the ventral receptacle. The latter potentially plays a crucial role in post-copulatory sexual selection as the likely site of fertilization. With the baseline data provided in this study, experimental studies can now be designed to investigate the significance of the ventral receptacle in female choice and its possible influence on sperm competition. Also, there are an increasing number of empirical studies that document the coevolution between male and female reproductive characters. We strongly suggest that sepsids are an ideal group to study the coevolution because the female structures are now known and are diverse.

## Authors' contributions

NP and MK performed the morphological preparations, the microscopic examinations and assembled the morphological character matrix. NP and RM conducted all of the phylogenetic analyses. All three authors participated in the design of the study and the writing of the manuscript. All authors have read and approved the final manuscript.

## Supplementary Material

Additional file 1 **Character and character state descriptions for morphological matrix of female reproductive tract**. Click here for file

Additional file 2 **Table of convergence estimates for female morphology, *COI *barcode and sepsid mating behavior**. Click here for file

## References

[B1] Takami Y Sota T Rapid diversification of male genitalia and mating strategies in *Ohomopterus *ground beetles Journal of Evolutionary Biology 2007 20 4 1385 1395 10.1111/j.1420-9101.2007.01338.x 17584233

[B2] Routto J Mazzi D Van Der Linde K Mirol P Butlin RK Hoikakala A The extent of variation in male song, wing and genital characters among allopatric *Drosophila montana *populations Journal of Evolutionary Biology 2007 20 4 1591 1601 10.1111/j.1420-9101.2007.01323.x 17584251

[B3] Bertin A Fairbairn DJ One tool, many uses: precopulatory sexual selection on genital morphology in *Aquarius remigis* Journal of Evolutionary Biology 2005 18 4 949 961 10.1111/j.1420-9101.2005.00913.x 16033567

[B4] Bernstein S Bernstein R Allometry of male genitalia in a species of soldier beetle: Support for the one-size-fits-all hypothesis Evolution 2002 56 8 1707 1710 12353764 10.1111/j.0014-3820.2002.tb01483.x

[B5] Lachmann AD Function and coevolution of male and female genitalia in tephritid fruit flies (Diptera, Tephritidae) Zoology (Jena) 2001 103 Supplement 3 46

[B6] Eberhard WG Male-female conflict and genitalia: failure to confirm predictions in insects and spiders Biological Reviews 2004 79 1 121 186 10.1017/S1464793103006237 15005176

[B7] Arnqvist G Rowe L Correlated evolution of male and female morphologies in water striders Evolution 2002 56 5 936 947 12093029 10.1111/j.0014-3820.2002.tb01406.x

[B8] Roenn J Katvala M Arnqvist G Coevolution between harmful male genitalia and female resistance in seed beetles Proceedings of the National Academy of Sciences of the United States of America 2007 104 26 10921 10925 10.1073/pnas.0701170104 17573531 PMC1904142

[B9] Hosken DJ Stockley P Sexual selection and genital evolution Trends in Ecology & Evolution 2004 19 2 87 93 10.1016/j.tree.2003.11.012 16701234

[B10] Eberhard WG Copulatory courtship and morphology of genitalic coupling in seven *Phyllophaga *species (Coleoptera: Melolonthidae) Journal of Natural History 1993 27 3 683 717 10.1080/00222939300770401

[B11] Jagadeeshan S Singh RS A time-sequence functional analysis of mating behaviour and genital coupling in *Drosophila*: role of cryptic female choice and male sex-drive in the evolution of male genitalia Evolutionary Biology 2006 19 1058 1070 10.1111/j.1420-9101.2006.01099.x 16780507

[B12] Eberhard WG Sexual Selection and Animal Genitalia Eberhard, W G Sexual Selection and Animal Genitalia Xii + 244p Harvard University Press: Cambridge, Mass, USA; London, England Illus 1985

[B13] Huber BA Mating positions and the evolution of asymmetric insect genitalia Genetica 2010 138 19 25 10.1007/s10709-008-9339-6 19089587

[B14] Song HJ Species-specificity of male genitalia is characterized by shape, size, and complexity Insect Systematics & Evolution 2009 40 2 159 170

[B15] House CM Simmons LW The evolution of male genitalia: patterns of genetic variation and covariation in the genital sclerites of the dung beetle *Onthophagus taurus* Journal of Evolutionary Biology 2005 18 1281 1292 10.1111/j.1420-9101.2005.00926.x 16135123

[B16] Joly D Schiffer M Coevolution of male and female reproductive structures in *Drosophila* Genetica 138 1 105 118 10.1007/s10709-009-9392-9 19657593

[B17] Arthur BI Sbilordo SH Pemberton AJ Ward PI The anatomy of fertilization in the yellow dung fly *Scathophaga stercoraria* Journal of Morphology 2008 269 5 630 637 10.1002/jmor.10617 18196572

[B18] Briceno RD Eberhard WG Robinson AS Copulation behaviour of *Glossina pallidipes *(Diptera : Muscidae) outside and inside the female, with a discussion of genitalic evolution Bulletin of Entomological Research 2007 97 471 488 10.1017/S0007485307005214 17916266

[B19] Twig E Yuval B Function of multiple sperm storage organs in female Mediterranean fruit flies (*Ceratitis capitata*, Diptera : Tephritidae) Journal of Insect Physiology 2005 51 1 67 74 10.1016/j.jinsphys.2004.11.007 15686648

[B20] Huelsenbeck JP Larget B Miller RE Ronquist F Potential applications and pitfalls of Bayesian inference of phylogeny Systematic Biology 2002 51 5 673 688 10.1080/10635150290102366 12396583

[B21] Hosken DJ Meyer EP Ward PI Internal female reproductive anatomy and genital interactions during copula in the yellow dung fly, *Scathophaga stercoraria *(Diptera : Scathophagidae) Canadian Journal of Zoology-Revue Canadienne De Zoologie 1999 77 12 1975 1983 10.1139/cjz-77-12-1975

[B22] Minder AM Hosken DJ Ward PI Co-evolution of male and female reproductive characters across the Scathophagidae (Diptera) Journal of Evolutionary Biology 2005 18 1 60 69 10.1111/j.1420-9101.2004.00799.x 15669961

[B23] Ward PI Cryptic female choice in the yellow dung fly *Scathophaga stercoraria *(L.) Evolution 2000 54 5 1680 1686 11108595 10.1111/j.0014-3820.2000.tb00712.x

[B24] Eberhard W Sexually antagonistic coevolution in insects is associated with only limited morphological diversity Journal of Evolutionary Biology 2006 19 3 657 681 10.1111/j.1420-9101.2005.01057.x 16674564

[B25] Hellriegel B Ward PI Complex female reproductive tract morphology: Its possible use in postcopulatory female choice Journal of Theoretical Biology 1998 190 2 179 186 10.1006/jtbi.1997.0546

[B26] Pitnick S Markow T Spicer GS Evolution of multiple kinds of female sperm-storage organs in *Drosophila* Evolution 1999 53 6 1804 1822 10.2307/2640442 28565462

[B27] Otronen M Male asymmetry and postcopulatory sexual selection in the fly *Dryomyza anilis* Behavioral Ecology and Sociobiology 1998 42 3 185 191 10.1007/s002650050430

[B28] Fritz AH Turner FR A light and electron microscopical study of the spermathecae and ventral receptacle of *Anastrepha suspensa *(Diptera : Tephritidae) and implications in female influence of sperm storage Arthropod Structure & Development 2002 30 4 293 313 10.1016/s1467-8039(01)00038-x 18088963

[B29] Manier MK Belote JM Berben KS Novikov D Stuart WT Pitnick S Resolving Mechanisms of Competitive Fertilization Success in *Drosophila melanogaster* Science 2010 328 5976 354 357 10.1126/science.1187096 20299550

[B30] Hellriegel B Bernasconi G Female-mediated differential sperm storage in a fly with complex spermathecae, *Scatophaga stercoraria* Animal Behaviour 2000 59 311 317 10.1006/anbe.1999.1308 10675253

[B31] Ilango K Structure and function of the spermathecal complex in the phlebotomine sandfly *Phlebotomus papatasi *Scopoli (Diptera : Psychodidae): II. Post-copulatory histophysiological changes during the gonotrophic cycle Journal of Biosciences 2005 30 5 733 747 10.1007/BF02703572 16388146

[B32] Ilango K Structure and function of the spermathecal complex in the phlebotomine sandfly *Phlebotomus papatasi *Scopoli (Diptera : Psychodidae): I. Ultrastructure and histology Journal of Biosciences 2005 30 5 711 731 10.1007/BF02703571 16388145

[B33] Eberhard WG Huber BA Copulation and sperm transfer in *Archisepsis *flies (Diptera, Sepsidae) and the evolution of their intromittent genitalia Studia Dipterologica 1998 5 217 248

[B34] Eberhard WG Courtship and multi-stage transfer of material to the female's wings during copulation in *Microsepsis armillata *(Diptera: Sepsidae) Journal of the Kansas Entomological Society 2001 74 2 70 78

[B35] Meier R Kotrba M Ferrar P Ovoviviparity and viviparity in the Diptera Biological Reviews 1999 74 3 Cambridge 199 258 10.1017/S0006323199005320

[B36] Lachmann AD Copulation and engagement of male and female genitalia in five *Coproica *Rondani species (Diptera: Sphaeroceridae) Annals of the Entomological Society of America 1996 89 5 759 769

[B37] Dufour L Recherches anatomiques et physiologiques sur les Diptères, accompagnées de considérations rélatives à l'historie naturalle de ces insectes Mémoires de l'Académie des Sciences de l'Institut de France 1851 11 171 630 plates 171-111

[B38] Sturtevant AH The seminal receptacles and accessory glands of the Diptera, with special reference to the Acalyptratae Journal of the New York Entomological Society 1925 33 4 195 215

[B39] Kiontke K Über die Bedeutung von Sepsiden (Diptera) und Sphaeroceriden (Diptera) für den Transport kuhfladenbewohnender Nematoden under besonderer Berücksichtung der Phoresie von *Diplogaster coprophilus *Sudhaus & Rehfled 1989 (Nematoda) Diplomarbeit des Fachbereiches Biologie der Freien Universität Berlin 1989

[B40] Kotrba M Das Reproduktionssystem von *Cyrtodiopsis whitei *Curran (Diopsidae, Diptera) unter besonderer Berucksichtigung der inneren weiblichen Geschlechtsorgane Bonner Zoologische Monographien 1993 33 1 106

[B41] Ozerov AL Mukhi-murav'evidki (Diptera, Sepsidae) fauny Rossii [The Sepsidae (Diptera) of Russia] Sbornik trudov Zoologicheskogo muzeya MGU [Archives of the Zoological Museum of Moscow State University] 2003 45 182

[B42] Ozerov AL World catalogue of the family Sepsidae (Insecta: Diptera) Zoologicheskie issledovania (Zoological Studies) 2005 8 1 74

[B43] Blanckenhorn WU Kraushaar URS Teuschl Y Reim C Sexual selection on morphological and physiological traits and fluctuating asymmetry in the black scavenger fly *Sepsis cynipsea* Journal of Evolutionary Biology 2004 17 3 629 641 10.1111/j.1420-9101.2004.00693.x 15149405

[B44] Eberhard WG Species-specific genitalic copulatory courtship in sepsid flies (Diptera, Sepsidae, *Microsepsis*) and theories of genitalic evolution Evolution 2001 55 1 93 102 11263749 10.1111/j.0014-3820.2001.tb01275.x

[B45] Hosken DJ Martin OY Born J Huber F Sexual conflict in *Sepsis cynipsea*: Female reluctance, fertility and mate choice Journal of Evolutionary Biology 2003 16 3 485 490 10.1046/j.1420-9101.2003.00537.x 14635848

[B46] Martin OY Hosken DJ Copulation reduces male but not female longevity in *Saltella sphondylli *(Diptera : Sepsidae) Journal of Evolutionary Biology 2004 17 2 357 362 10.1046/j.1420-9101.2003.00668.x 15009269

[B47] Puniamoorthy N Tan D Ismail M Meier R From kissing to belly stridulation: comparative analysis reveals surprising diversity, rapid evolution, and much homoplasy in the mating behavior of 27 species of sepsid flies (Diptera: Sepsidae) Journal of Evolutionary Biology 2009 22 2146 2156 10.1111/j.1420-9101.2009.01826.x 19732260

[B48] Ang Y Puniamoorthy N Meier R Secondarily reduced foreleg armature in *Perochaeta dikowi *sp. n. (Diptera: Cyclorrhapha: Sepsidae) due to a novel mounting technique Systematic Entomology 2008 33 552 559 10.1111/j.1365-3113.2008.00422.x

[B49] Ingram KK Laamanen T Puniamoorthy N Meier R Lack of morphlogical coevolution between male forelegs and female wings in *Themira *(Sepsidae: Dipera: Insecta) Biological Journal of the Linnean Society 2008 93 227 238 10.1111/j.1095-8312.2007.00922.x

[B50] Puniamoorthy N Su Feng Yi K Meier R Bending for love: losses and gains of sexual dimorphisms are strictly correlated with changes in the mounting position of sepsid flies (Sepsidae: Diptera) BMC Evolutionary Biology 2008 8 155 10.1186/1471-2148-8-155 18492287 PMC2409323

[B51] Su KFY Kutty S Meier R Morphology versus Molecules: The phylogenetic relationships of Sepsidae (Diptera: Cyclorrhapha) based on morphology and DNA sequence data from ten genes Cladistics 2008 24 902 916 10.1111/j.1096-0031.2008.00222.x 34892884

[B52] Meier R Cladistic analysis of the Sepsidae (Cyclorrhapha: Diptera) based on a comparative scanning electron microscopic study of larvae Systematic Entomology 1995 20 2 99 128 10.1111/j.1365-3113.1995.tb00086.x

[B53] Meier R A comparative SEM study of the eggs of the Sepsidae (Diptera) with a cladistic analysis based on egg, larval and adult characters Entomologica Scandinavica 1995 26 4 425 438

[B54] Meier R Larval morphology of the Sepsidae (Diptera: Sciomyzoidea), with a cladistic analysis using adult and larval characters Bullentin of the American Museum of Natural History 1996 228 1 147

[B55] Hebert PDN Ratnasingham S deWaard JR Barcoding animal life: Cytochrome c oxidase subunit 1 divergences among closely related species Proceedings of the Royal Society Biological Sciences Series B 2003 270 Supplement 1 S96 S99 10.1098/rsbl.2003.0025 PMC1698023 12952648

[B56] Meier R Kwong S Vaidya G Ng PKL DNA Barcoding and Taxonomy in Diptera: a Tale of High Intraspecific Variability and Low Identification Success Systematic Biology 2006 55 715 728 10.1080/10635150600969864 17060194

[B57] Andersson H A new species of *Themira *R.-D. from South Sweden (Diptera: Sepsidae) Entomologica Scandinavica 1975 6 57 60

[B58] Kotrba M Darvas LPB Morphology and terminology of the female postabdomen Contributions to a Manual of Palaeartic Diptera (with special reference to flies of economic importance) 2000 1 Budapest: Science Herald 75 84

[B59] Maddison DR Maddison WP MacClade 4: Interactive Analysis of Phylogeny and Character Evolution 2000 4.01 Sunderland, MA.: Sinauer Associates 10.1159/000156416 2606395

[B60] Swofford DL PAUP*: Phylogenetic analysis using parsimony (and other methods) 2004 4.0b10 Sunderland, Massachusetts: Sinauer Associates, Inc

[B61] Vaidya G Lohman DL Meier R SequenceMatrix: concatenation software for the fast assembly of multigene datasets with character set and codon information Cladistics 2010 10.1111/j.1096-0031.2010.00329.x 34875773

[B62] Meier R Kores P Darwin S Homoplasy slope ratio: A better measurement of observed homoplasy in cladistic analyses Systematic Zoology 1991 40 1 74 88 10.2307/2992223

[B63] De Carlo JM Pellerano GN Martinez LI Saco del oviducto medio de *Ceratitis capitata *Wied. (Diptera: Tephritidae): consideraciones histo-funcionales Physis 1994 49 19 25

[B64] Perez-Staples D Harmer AMT Taylor PW Sperm storage and utilization in female Queensland fruit flies (*Bactrocera tryoni*) Physiological Entomology 2007 32 2 127 135 10.1111/j.1365-3032.2006.00554.x

[B65] Meier R Zhang G Ali F The use of mean instead of smallest interspecific distances exaggerates the size of the "barcoding gap" and leads to misidentification Systematic Biology 2008 57 809 813 10.1080/10635150802406343 18853366

[B66] House CM Simmons LW Genital morphology and fertilization success in the dung beetle *Onthophagus taurus*: An example of sexually selected male genitalia Proceedings of the Royal Society Biological Sciences Series B 2003 270 1514 447 455 10.1098/rspb.2002.2266 PMC1691274 12641898

[B67] Eberhard W Evolution of genitalia: theories, evidence, and new directions Genetica 138 1 5 18 10.1007/s10709-009-9358-y 19308664

[B68] Arnqvist G Rowe L Sexual conflict 2005 Princeton: Princeton University Press

[B69] Rowe L Westlake KP Currie DC Functional significance of elaborate secondary sexual traits and their evolution in the water strider genus *Rheumatobates* Canadian Entomologist 2006 138 4 568 577 10.4039/N06-811

[B70] Morrow EH Arnqvist G Costly traumatic insemination and a female counter-adaptation in bed bugs Proceedings of the Royal Society Biological Sciences Series B 2003 270 1531 2377 2381 10.1098/rspb.2003.2514 PMC1691516 14667354

[B71] Bergsten J Miller KB Phylogeny of diving beetles reveals a coevolutionary arms race between the sexes PLoS ONE 2007 2 6 10.1371/journal.pone.0000522 17565375 PMC1885976

[B72] Martin OY Hosken DJ The evolution of reproductive isolation through sexual conflict Nature 2003 423 6943 979 982 10.1038/nature01752 12827200

[B73] Martin OY Hosken DJ Costs and benefits of evolving under experimentally enforced polyandry or monogamy Evolution 2003 57 12 2765 2772 14761055 10.1111/j.0014-3820.2003.tb01518.x

[B74] Eberhard WG Female control: Sexual selection by cryptic female choice 1996 Princeton: Princeton University Press

[B75] Miller GT Pitnick S Sperm-female coevolution in *Drosophila* Science 2002 298 5596 1230 1233 10.1126/science.1076968 12424377

[B76] Snook RR Hosken DJ Sperm death and dumping in *Drosophila* Nature 2004 428 6986 939 941 10.1038/nature02455 15118726

[B77] Otronen M Fertilisation success in the fly *Dryomyza anilis *(Dryomyzidae): Effects of male size and the mating situation Behavioral Ecology and Sociobiology 1994 35 1 33 38 10.1007/BF00167057

[B78] Dallai R Marchini D Del Bene G The ultrastructure of the spermathecae in *Ceratitis capitata *Wied. and *Dacus oleae *Gmel (Diptera: Tephritidae) Redia 1993 76 147 167

[B79] Solinas M Nuzzaci G Functional anatomy of *Dacus oleae *Gmel. female genitalia in relation to insemination and fertilization processes Entomologica 1984 19 135 165

[B80] Fritz AH Sperm storage patterns in singly mated females of the Caribbean fruit fly, *Anastrepha suspensa *(Diptera : Tephritidae) Annals of the Entomological Society of America 2004 97 6 1328 1335 10.1603/0013-8746(2004)097[1328:SSPISM]2.0.CO;2

[B81] Kotrba M Mathis WN The internal female reproducive tract of the enigmatic genus *Risa *(Diptera: Schizophora: Ephydroidea) and its phylogenetic implications Proceedings of the Entomological Society of Washington 2009 111 3 627 640

[B82] Kotrba M Baptista AR The internal reproductive tract of Opomyzidae (Diptera, Schizophora) Studia Dipterologica 2002 9 1 57 71

