# Supplementary material for: Unlocking the "Black box": internal female genitalia in Sepsidae (Diptera) evolve fast and are species-specific
Source: BMC Evol Biol. 2010 Sep 10;10:275. doi: 10.1186/1471-2148-10-275 (PMC2944183; doi:10.1186/1471-2148-10-275)
Supplement: Additional file 1 — Character and character state descriptions for morphological matrix of female reproductive tract. [file 1471-2148-10-275-S1.DOC]

**Detailed description of the nineteen female morphological characters and their respective character states.**

**1. Antero-dorso differentiation of ventral receptacle wall** Unordered; Binary

0: Not enlarged

1: Enlarged

**2. Sclerotization of ventral receptacle** Unordered; Multistate

0: Not sclerotized

1: Antero-dorso portion of wall slightly sclerotized

2: Antero-dorso portion of wall completely sclerotized

3: Chambers are completely sclerotized

4: Presence of a'diffused' sclerite

**3. Ratio of ventral evagination to ventral receptacle** Ordered; Multistate

0: 0.5

1: 0.8

2: 1.0

3: 1.5

4: 2.0

5: 2.5

6: 3 to 4

**4. Shape of Ventral receptacle** Unordered; Multistate

0: Tubular

1: Bilobed

2: Multichambered

**5. No. of chambers (Multichambered)** Ordered; Multistate

0: 10

1: 13

2: 16

3: 17

4: 19

5: 20

6: 27

7: 32

8: 80

**6. No. of subdivisions (bilobed)** Ordered; Multistate

0: 0

1: 4

2: 6

3: 7

4: 9 chambers

5: 10

6: 12 chambers

**7. Base of ventral receptacle** Unordered; Binary

0: Short

1: Long stalk

**8. Presence of dorsal sclerite** Unordered; Binary

0: Structure absent

1: Dorsal sclerite present

**9. Presence of distinct apical process on dorsal sclerite** Unordered; Binary

0: Absent

1: Present

**10. Ornamentation on basal cheeks of dorsal sclerite** Unordered; Multistate

0: None

1: 'Dimples'

2: 'Honeycomb'

**11. Additional sclerotization in the dorsal vaginal wall (paired)** Unordered; Binary

0: Absent

1: Present

**12. Reduction of spermathecae**  Unordered; Binary

0: Present

1: Reduced

**13. Base of spermathecae** Unordered; Binary

0: Not or slightly telescoped

1: Deeply telescoped

**14. Opening of spermathecal ducts into vagina** Unordered; Multistate

0: Separately

1: Fused

2: Fused and strongly sclerotized

**15. Size of spermathecae** Unordered; Binary

0: Equal

1: Distinctly unequal

**16. Length of spermathecal ducts** Unordered; Binary

0: Equal

1: Distinctly unequal

**17. Posterior part of Sternite VII** Unordered; Binary

0: Bifurcated

1: Fused

**18. Transverse folds in anterior part of inverted ovipositor** Unordered; Binary

0: Absent

1: Present

**19. Arrangement of spines on ovipositor** Unordered; Multistate

0: Spines absent

1: Large, single spines evenly spaced

2: Long, thin, single spines evenly spaced

3: Large, single spines in irregular rows

4: Small spines in tight clusters

5: Tiny spines in regular rows

6: Large, regular and tightly packed spines

7: Long, thin spines in dense rows

8: Small spines in regular rows
